# Association Between Abundance of *Haemophilus* in the Gut Microbiota and Negative Symptoms of Schizophrenia

**DOI:** 10.3389/fpsyt.2021.685910

**Published:** 2021-07-30

**Authors:** Cuizhen Zhu, Mingming Zheng, Usman Ali, Qingrong Xia, Zhongxian Wang, Chenlong, Lihui Yao, Yuanyuan Chen, Junwei Yan, Keming Wang, Jinghong Chen, Xulai Zhang

**Affiliations:** ^1^Department of Science and Education, Affiliated Psychological Hospital of Anhui Medical University, Hefei, China; ^2^Anhui Clinical Center for Mental and Psychological Diseases, Hefei Fourth People's Hospital, Hefei, China; ^3^Anhui Mental Health Center, Hefei, China; ^4^Shanghai key Laboratory of Psychotic Disorders, Shanghai Mental Health Center, Shanghai Jiao Tong University School of Medicine, Shanghai, China

**Keywords:** schizophrenia, gut microbiota, 16Sr RNA sequencing, negative symptom, positive and negative syndrome scale

## Abstract

Increasing evidence indicates an interaction between dysbiosis of the microbiota and the pathogenesis of schizophrenia. However, limited information is available on the specific microbial communities associated with symptoms of schizophrenia. Therefore, this study aimed to investigate gut microbiota dysbiosis and its relationship with psychopathologies in schizophrenia. We recruited 126 participants and divided them into three groups according to the Diagnostic and Statistical Manual of Mental Disorders, Fifth Edition, criteria—acute group (patients with acute schizophrenia), remission group (patients with schizophrenia in remission), and control group (healthy controls). Psychotic symptoms were evaluated using the Positive and Negative Syndrome Scale. Microbiota compositions, diversity and community structure were evaluated using 16S rRNA sequencing. Pearson's correlation analysis was used to evaluate the association between bacterial taxa and psychotic symptoms. The beta-diversity of microbiota composition in the acute group was distinct from that in the remission and control groups (PC1 = 21.11% vs. PC2 = 12.86%, *P* = 0.021). Furthermore, Pearson's correlation analysis revealed that abundance of *Haemophilu*s was positively correlated with negative psychiatric symptoms (*r* = 0.303, *P* = 0.021), while abundance of *Coprococcus* was negatively correlated with negative psychiatric symptoms (*r* = −*0.285, P* = *0.025*). Moreover, abundance of *Haemophilus* was positively correlated with cognition (*r* = 0.428, *P* = 0.009), excitement (*r* = 0.266, *P* = 0.037), and depression (*r* = 0.295, *P* = 0.020). The study findings suggest that alterations in certain gut microbiota may interfere with psychological symptoms in schizophrenia. Our results provide evidence that may help in the development of therapeutic strategies using microbial-based targets. The data that support the findings of this study have been deposited in the NCBI (https://submit.ncbi.nlm.nih.gov/) with accession number SUB9453991.

## Introduction

Schizophrenia is a severe psychiatric disorder that significantly affects patients' quality of life (1, 2). It is a complicated genetic disorder of unknown aetiology that is prevalent in ~1% of the general population. However, the biological mechanisms of the disease are relatively unknown (3, 4). The mean age range of onset of schizophrenia is 15–35 years (5). Schizophrenia is characterised by multifactorial aetiopathogenesis, which is a key research field. Patients with schizophrenia may present with positive symptoms, such as hallucinations, delusions, disorganised behaviour, and negative symptoms, including absence of interest and motivation, blunted affect, asociality, and avolition. Negative symptoms are the core component of schizophrenia, leading to a large proportion of mental disability and long-term dysfunction (6–8). Negative symptoms seriously damage the patients' social function and quality of life. Importantly, negative symptoms are also potential predictors of psychosis conversion. The current knowledge on the aetiology, pathology, and effective treatment of these negative symptoms is limited. Further studies are warranted to determine the exact pathophysiology of the disease (9, 10).

The intestinal flora alters a variety of physiological and pathophysiological processes in the body (11, 12). Recent evidence suggests that the microbes residing in the human intestine are considered critical influencers of mental health and play an important role in various neuropsychological disorders, including anxiety (13), depression (14), fibromyalgia (15), schizophrenia (16), sleep disorders (17, 18), Parkinson's disease (19), and Alzheimer's disease (20). Previous studies have stated that the interactions between the gut microbiota and the brain are bi-directional. Gut microbes modulate the inter-communication among the nervous, endocrine, and immune systems in the brain–gut axis. The nervous system disrupts the intestinal system *via* secretion of different biochemicals, such as γ-aminobutyric acid, dopamine, norepinephrine and serotonin (21, 22). Schizophrenia is a devastating psychiatric disorder that involves emotional, occupational, and cognitive impairment (23, 24). Treatment protocols for schizophrenia can be classified as psychosocial rehabilitation or pharmacotherapy. However, these therapies are not beneficial for many patients, it is challenging to treat schizophrenia (25, 26). The gut microbiota plays an important role in schizophrenia, therefore, the effects of gut microbiota on schizophrenia require further investigation, especially regarding the mechanism of inter-communication (3).

Previous studies have found a vast dysbiosis in the intestinal flora between patients with schizophrenia and healthy individuals (23, 27). Behavioural changes have been found in healthy subjects who were administered with the gut microbiota of patients with schizophrenia (28, 29). Therefore, there is a need to identify new strategies targeting the gut microbiota to improve the diagnosis and treatment of schizophrenia. Accordingly, we hypothesised that intestinal dysbiosis results in exacerbation of schizophrenia. In this study, we investigated the composition of the faecal microbiota of patients at different stages of schizophrenia (acute vs. remission) and compared it to that of healthy subjects using the Positive and Negative Syndrome Scale (PANSS). We also explored how the detected compositional dysbiosis were associated with symptom severity and remission. Moreover, this study provided insights into the association among the gut microbiota composition of schizophrenia at different stages involving a single symptom or multiple symptoms.

## Methods

### Ethics Statement

The study was approved by the Medical Ethics Committee of the Anhui Mental Health Centre (AMHC). All participants provided written consent prior to study participation in accordance with the principles of the Declaration of Helsinki. The trial clinical registration number was chiCTR1800019343.

### Procedure

**Figure 1:**
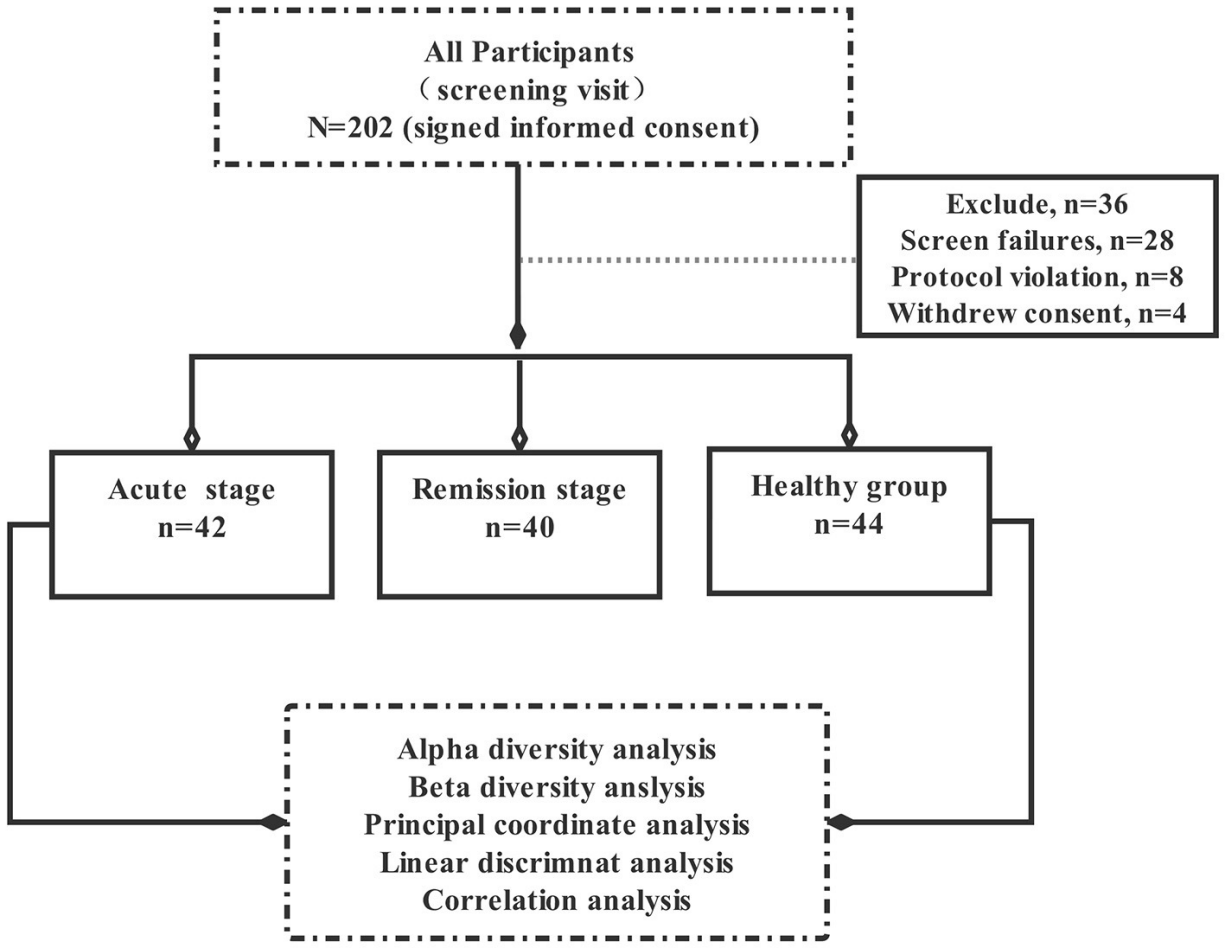
Study selection process.

This pilot study explored the relationship between intestinal flora imbalance and schizophrenia symptoms. A total of 202 participants were initially selected. Of these, 36 participants did not meet the inclusion criteria, 28 individuals could not complete the scale assessment, eight people who took antibiotics, and four participants refused to sign informed consent, Hence, total of 76 subjects who did not meet the experimental criteria were excluded from this experiment. Ultimately, the remaining 126 participants were included in this study and divided them into three groups according to the Diagnostic and Statistical Manual of Mental Disorders, Fifth Edition (DSM-5), —acute group (patients with acute schizophrenia, *n* = 42), remission group (patients with schizophrenia in remission, *n* = 40), and control group (healthy controls, *n* = 44). All patients were hospitalised at the AMHC between January 2018 and September 2020, healthy controls are the healthy people recruited by the hospital physical examination centre (Figure 1).

All participants were assessed using the Mini-International Neuropsychiatric Interview (MINI) 6.0.0. According to the trial standards, patients of acute group and remission group diagnosed with schizophrenia met the criteria of the DSM-5. The inclusion criteria for the patients in the acute group were as follows: (1) age = 18–60 years, (2) fulfilment of the DSM-5 criteria for schizophrenia, (3) the first episode of schizophrenia without anti-psychotic treatment, and (4) PANSS total score ≥60 points. The inclusion criteria for the patients in the remission group were as follows: (1) fulfilment of the first and second criteria of the acute group, (2) patients with a total course of disease <10 years, received only second-generation anti-psychotic, such as risperidone, quetiapine, and aripiprazole, (3) the patient's clinical symptoms disappeared after treatment and self-consciousness and social function had recovered for at least 3 months, and (4) PANSS total score <60 points. Healthy controls were recruited from the local community at the same time as other participants through advertising. The exclusion criteria were as follows: (1) a history of craniocerebral trauma, organic cerebral diseases, physical diseases, or other mental disorders; (2) a history of alcohol or other substance use; (3) diabetes, hypertension, dyslipidemia, endocrine disease, or known medical conditions that might affect metabolism; (4) pregnant or lactating women; (5) a history of digestive tract diseases, abdominal surgery, or intestinal infection in the past 3 months; (6) administration of electroconvulsive therapy without convulsions before enrolment;(7) diet change significantly in the past 6 months; (8) treatment with antibiotics or corticosteroids, probiotic preparations, or other immune preparations in the past 3 months.

### Clinical Assessments

#### MINI 6.0.0

Participants were screened by experienced psychiatrists for inclusion in the study. The initial clinical diagnoses were validated using the MINI 6.0.0. It is a concise diagnostic interview for psychiatric disorders developed jointly by psychiatrists in the United States and Europe. It has been structured to meet the criteria for a brief psychiatric interview that can be conducted in 15 min. All patients underwent the MINI 6.0.0 to confirm the clinical diagnosis of schizophrenia (30).

#### PANSS

The PANSS is a widely used instrument for measuring severe psychopathology in adult patients with schizophrenia. It has been utilised for the assessment of positive and negative symptoms, as shown in **Table 2**. A Mandarin version of a five-factor model of the PANSS has been proven to have good reliability and validity, with a Cronbach's alpha coefficient of 0.928 and an intra-class coefficient of 0.878 (31).

### Stool Sample Collection, Storage, and Processing

Stool samples were collected by trained nurses using a sample collection protocol. All samples were transferred to the laboratory, where they were mechanically homogenised with a sterile spatula, aliquoted into sterile 2-mL cryovials, and stored in a freezer at −80°C for future DNA isolation and nucleotide sequencing. Microbial DNA concentrations in the faeces were determined by amplification of the bacterial 16S rRNA genes and were extracted using the Qiagen QIAamp Fast DNA stool MINI Kit according to the manufacturer's instructions. The V3–V4 hypervariable region of the 16S rRNA gene was amplified. All selected DNA segments were sequenced in the paired-end mode using Illumina HiSeq2500 (Huada Genomics Technology Service Co., Ltd., Shenzhen, China).

### Data Analysis

#### Demographic Analysis

The SPSS, version 22.0, statistical package for Windows (IBM Corp., USA) was used for data analysis. Demographic data and clinical characteristics were compared among the three groups. Continuous variables were analysed using an independent samples *t*-test, and discontinuous variables were compared using the χ^2^ test.

#### Microbiome Analyses

To explore the diversity of the gut microbiota, we performed R (version 3.3.2) and R Studio (version 1.0.136) to calculate a range of estimators. Principal coordinate analysis (PCA) based on the Unweighted UniFrac distance matrix was used to identify differentially abundant operational taxonomic units. A linear discriminant analysis effect size (LEfSe) procedure was used with the Kruskal–Wallis rank-sum test to identify microbiome biomarkers in the faecal samples from the three groups at various taxonomic ranks. Bacteria with a linear discriminant analysis score of 2 were defined as significantly abundant. For the construction of the heat map, log_10_ fold change ratios from medians were calculated from the group medians of highly abundant bacteria at the genus level. Pearson's correlation analysis was used to identify the relationship between dysbiosis intestinal flora at the genus level and psychiatric symptoms. For all statistical analyses, *P* < 0.05 (two tailed) was considered significant.

## Results

### Clinical Characteristics of Study Participants

The baseline demographic and clinical characteristics of the study participants are presented in Table 1. In total, 126 participants were included in the final analyses. Of these participants, 44 participants were included in the healthy control group, and the others were divided into the acute and remission groups depending on the disease stage. There were no statistically significant differences among the groups in terms of baseline demographic and clinical characteristics such as age, sex, body mass index, and year of schooling. All patients underwent strict PANSS assessments by experienced psychiatrists. The decrease in PANSS total scores from baseline was significantly different between the acute group and remission group (29.8 ± 12.3 vs. 10.3 ± 4.9, *P* = 0.007).

**Table 1:** Baseline characteristics of the three groups.

**Characteristics**	**Acute stage**	**Remission stage**	**Healthy group**	***F*//χ^2^**	***P***
**Variable**	***n* = 42**	***n* = 40**	***n* = 44**		
Sex (F/M)	15/17	14/16	18/16	1.849	0.163
Age (years)	39.8 ± 11.7	41.1 ± 11.0	42.1 ± 10.4	1.356	0.701
BMI (kg/m^2^)	22.3 ± 3.4	24.3 ± 4.1	24.3 ± 4.1	2.432	0.181
Years of education	9.7 ± 4.4	9.9 ± 4.0	10.1 ± 4.6	1.103	0.902
PANSS scores	29.8 ± 12.3	10.3 ± 4.9	/	2.589	0.007

*F, Female; M, male; BMI, Body mass index; PANSS, Positive and negative syndrome*.

### Diversity Alteration in Faecal Microbiota Composition Within the Three Groups

**Figure 2:**
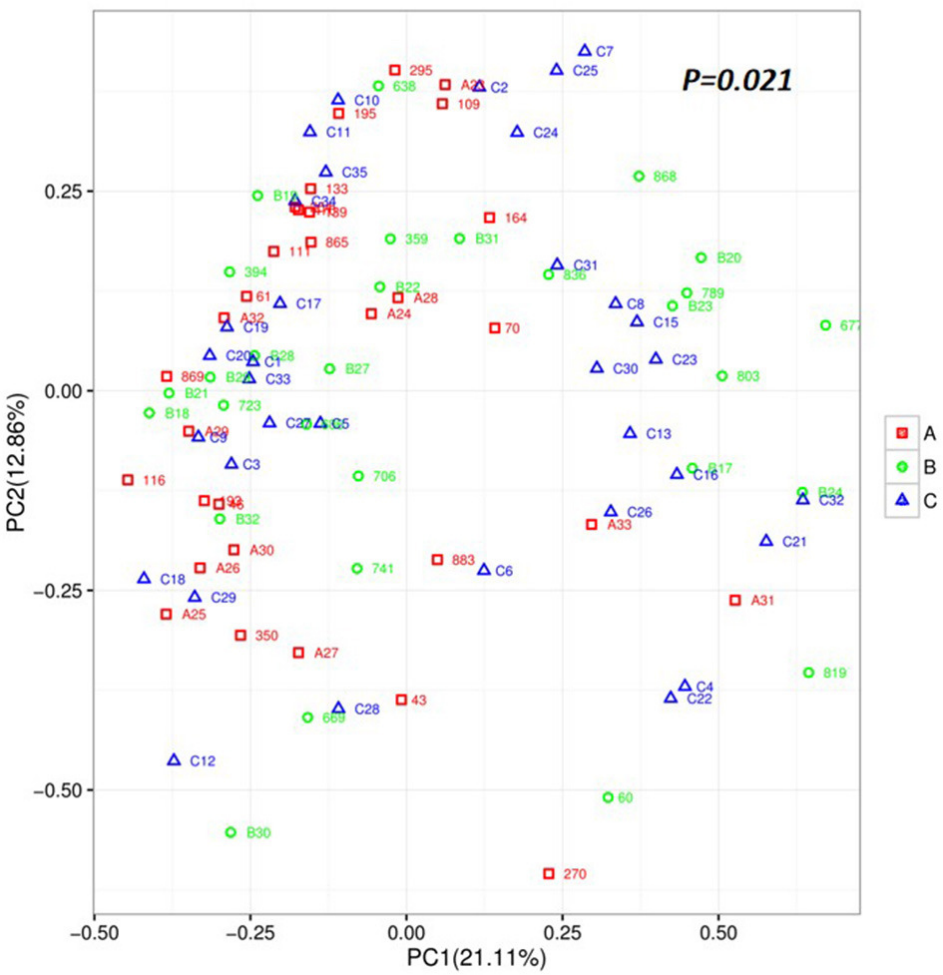
Unweighted based on the distance matrix of UniFrac dissimilarity of the faecal microbial communities in the Acute set stage group, Remission stage group, and Healthy control groups. Respective *R*-values show the community variation between the compared groups, and significant *P*-values are indicated. Acute stage group (red), Remission stage group (green), and Healthy group (blue). PC, principal coordinate.

To explore the richness and diversity of the bacterial community within the acute, remission, and healthy groups, we calculated the alpha and beta metrics. The alpha-diversity was not different among the three groups (Supplementary Figure 1). For beta-diversity, we used PCA ordination of unweighted UniFrac distances (Figure 2) to display the discrepancy among the three groups. We found that the gut microbiota composition of the acute group was different from that of the remission and control groups (PC1 = 21.11% vs. PC2 = 12.86%, *P* = 0.021). The results of beta-diversity analyses demonstrate that the overall composition of the microbiota in acute group has changed obviously compared with remission and healthy groups.

### Differentially Abundant Bacterial Taxa of the Acute, Remission, and Healthy Control Groups

**Figure 3:**
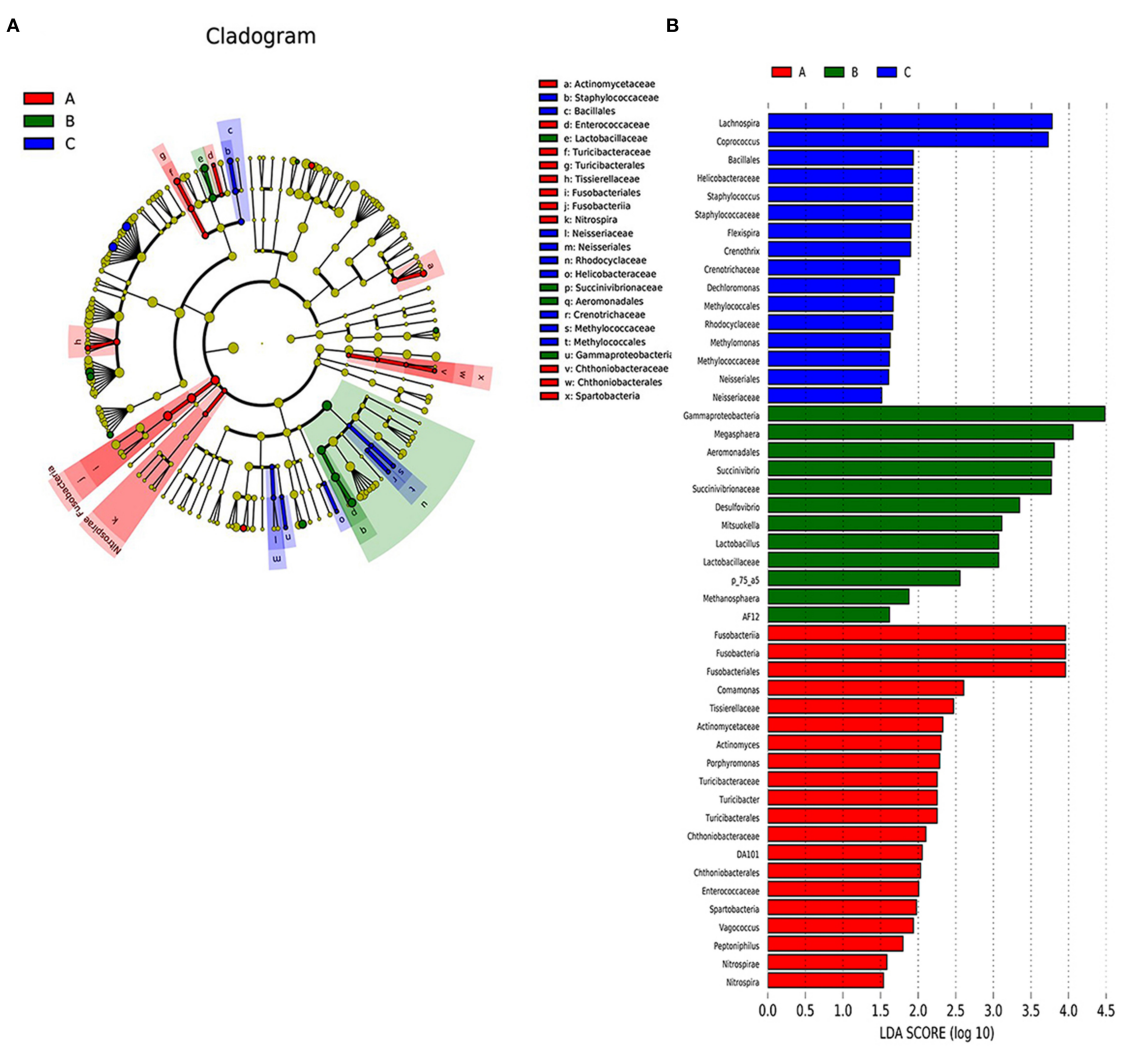
The differently abundant taxa identified using LEfSe analysis. **(A)** LEfSe clagogram showed the most differentially abundant taxa among the groups. Taxa enriched for health group in blue. Acute set stage group enriched taxa in red. Remission group enriched taxa in green. The size of each dot is proportional to its effect size. **(B)** Visualisation of only taxa meeting an LDA threshold >2. Taxa with enriched levels in onset stage group are shown in red, green represented taxa with enriched levels in remission stage group, blue represented taxa with enriched levels in health group.

To identify the types of bacterial taxa that varied among the three groups, we employed LEfSe analysis, an algorithm for high-dimensional biomarker discovery, which uses LDA >2 to estimate the effect size of each taxon differentially represented in the three groups. LEfSe (Figure 3B) identified 48 discriminative features whose relative abundances varied significantly among the acute, remission, and healthy control groups. At the genus level, the faecal microbiota of the acute group was differentially enriched with the genera *Fusobacteriales, Actinomyces, Turicibacter, Turicibacterales*, and *Chthoniobacterales*, while the healthy control group was enriched with *Lachnospira* and *Coprococcus*. At the order level, two microbiota members *Succinivibrionaceae* and *Lactobacillaceae* displayed an enriched trend in the remission group. Five microbiota members, *Tissierellaceae, Turicibacteraceae, Chthoniobacteraceae, Actinomycetaceae*, and *Enterococcaceae* were identified in the onset group. At the phylum level, we found that four microbiotas, *Desulfovibrio, Mitsuokella, Lactobacillus*, and *Succinivibrio*, were enriched in the remission group. The cladogram (Figure 3A) showed the structure of the faecal microbiota and the predominant bacteria in the acute, remission, and healthy control groups, revealing that the most significant shifts in the composition of the microbiota were primarily related to *Fusobacteria, Nitrospira, Succinivibronaceae*, and *Methylococcaceae*.

### Exploring the Relationship Between Dysbiosis Markers at the Genus Level and Psychotic Symptoms

**Figure 4:**
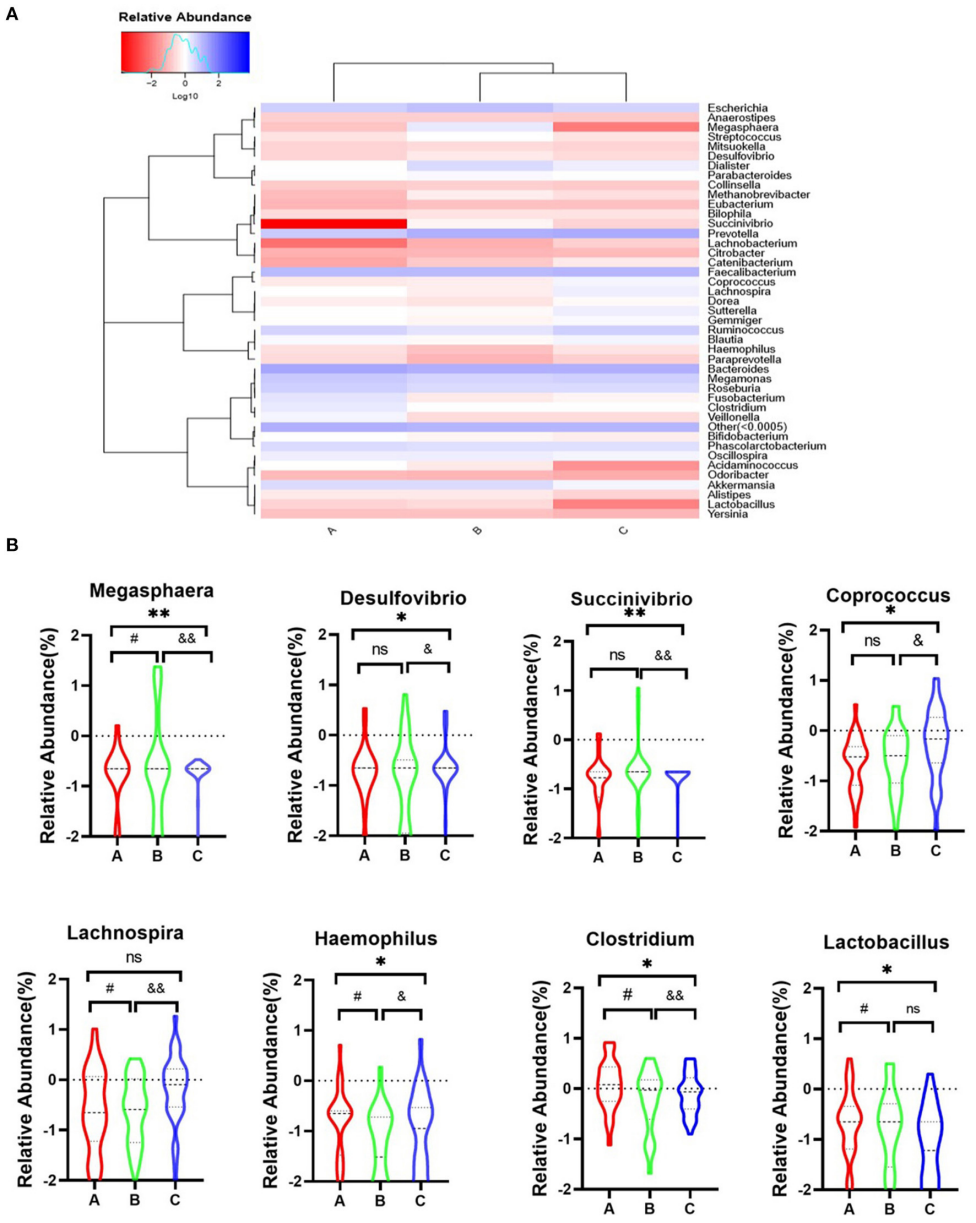
Heatmap representation of genus level of gut microbiota in three groups. **(A)** Heatmap of the 42 most counts abundant taxa. On top of the heatmap, group of samples are colour-coded. **(B)** A, Acute set stage group; B, Remission stage group; C, Healthy control group. *P_*A*−*C*_ ≤ 0.05, **P_*A*−*C*_ ≤ 0.001, ^#^*P*_*A*−*B*_ ≤ 0.05, ^&^*P*_*B*−*C*_ ≤ 0.05, and ^&&^*P*_*B*−*C*_ ≤ 0.001.

Further analysis will focus on the relationship between dysbiosis markers of microbio and psychotic symptoms among three groups. The genus-level analysis identified 42 genera in the three groups (Figure 4A). We observed eight markers at the genus level, which showed significant changes among the three groups (Figure 4B), including *Megasphaera* [*F*_(2, 90)_ = 11.72, *P* = 0.001], *Desulfovibrio* [*F*_(2, 88)_ = 3.940, *P* = 0.023], *Succinivibrio* [*F*_(2, 91)_ = 6.190, *P* = 0.003], *Coprococcus* [*F*_(2, 86)_ = 3.670, *P* = 0.003], *Lachnospira* [*F*_(3, 88)_ = 4.279, *P* = 0.016], *Haemophilus* [*F*_(3, 82)_ = 4.840, *P* = 0.010], *Clostridium* [*F*_(3, 78)_ = 2.655, *P* = 0.047], and *Lactobacillus* [*F*_(3, 88)_ = 2.575, *P* = 0.017]. *Post-hoc* Tukey's multiple comparison test further assessed the differences in these eight markers among the three groups. The results showed a high abundance of *Coprococuus* and a low abundance of *Megasphaera, Succinivibrio*, and *Clostridium* in the healthy group. The *Megasphaera* and *Megamonas* numbers was high in the remission group, while those of *Haemophilus* and *Faecalibacterium* were low in the acute group.

Previously, correlation analysis has revealed that the structures of the bacterial community are related to disease symptoms. To assess the association between these dysbiosis markers and psychotic symptoms, we performed Pearson's correlation analysis for these dysbiosis markers with five factors of the PANSS (Table 2). The results showed that abundance of *Haemophilus* was positively associated with negative psychiatric symptoms (*r* = 0.303, *P* = 0.021), while abundance of *Coprococcus* was negatively associated with negative psychiatric symptoms (*r* = −0.285, *P* = 0.025). Moreover, abundance of *Haemophilus* was positively associated with cognition (*r* = 0.428, *P* = 0.009), excitement (*r* = 0.266, *P* = 0.037), and depression (*r* = 0.295, *P* = 0.020).

**Table 2:** Correlation analysis between bacterial genus and PANSS.

**Bacteria genus**	**PANSS**											
	**Positive**		**Negative**		**Cognition**		**Excitement**		**Depression**		**Total**	
	***R***	***P***	***R***	***P***	***R***	***P***	***R***	***P***	***R***	***P***	***R***	***P***
Succinivibrio	0.023	0.857	0.010	0.940	0.061	0.637	0.010	0.939	0.059	0.650	0.027	*0.836*
Haemophilus	0.189	0.142	0.303^*^	0.021	0.428^**^	0.009	0.266^*^	0.037	0.295^*^	0.020	0.274^*^	0.030
Lactobacillus	0.049	0.704	0.042	0.744	0.172	0.182	0.112	0.385	0.070	0.587	0.121	0.347
Megasphaera	−0.139	0.281	0.047	0.718	0.015	0.908	0.113	0.380	0.019	0.886	0.027	0.837
Desulfovibrio	−0.139	0.281	0.045	0.731	0.085	0.511	0.038	0.770	−0.077	0.550	0.017	0.899
Coprococcus	0.027	0.833	−0.285^*^	0.025	−0.090	0.488	0.069	0.592	−0.039	0.764	−0.135	0.297
Clostridum	0.170	0.186	0.196	0.127	0.186	0.149	0.045	0.731	−0.028	0.828	0.188	0.143
Lachnospira	0.135	0.296	−0.038	0.771	0.039	0.764	0.049	0.705	0.216	0.910	0.039	0.761

*Correlation analysis between bacterial genus and PANSS. Bacterial genera were selected from Figure 4 of the heatmap according to the following criteria: taxa that were significantly different from the healthy control group. Among those taxa, succinivibrio, Haemophilus, lactobacillus, megasphaera, desulfovibrio, coprococcus, clostridum, and lachnospira were chosen. Haemophilus is positive associated with psychiatric symptoms on PANSS, Coprococcus is negative associated with negative symptoms on PANSS. PANSS, Positive and negative syndrome scale*.

* *P ≤ 0.05*,

** *P ≤ 0.001*.

## Discussion

Mental health is ‘a state of well-being in which the individual realises his or her own abilities, can cope with the normal stresses of life, works productively and fruitfully, and makes a contribution to his or her community’ (32). The gut microbiota plays an essential role in the development of neuropsychological disorders. Recently, increasing evidence highlights the crucial role of the gut microbiota in neuropsychological diseases (13, 33). Previous studies have indicated that dysbiosis microbiota involve several pathological conditions including but not limited to anxiety (13), depression (14), fibromyalgia (15), sleep disorder (17), Parkinson's disease (19), and Alzheimer's disease (20). The human gut microbiota is dynamic depending on various factors such as diet, emotion, and disease state (34). Recent studies have revealed an association between gut microbiota and the development of schizophrenia, implying the potential of using gut microbiota as a novel target for research on schizophrenia (16, 35, 36). Despite these findings, studies exploring the association between specific gut microbiota and schizophrenia symptoms are scarce. Moreover, the exact relationship between these specific gut microbiota and schizophrenia symptoms is unclear. The floras associated with symptoms of schizophrenia and how these floras affect the symptoms of schizophrenia are unknown. More studies are needed to explore the causal relationship between the gut microbiota and schizophrenia symptoms. In this study, we sought to evaluate the effects of gut microbiota on schizophrenia. The results indicated a remarkable relationship between the gut microbiota and schizophrenia. We demonstrated diversity in the composition of the gut microbiota at different stages of schizophrenia.

We analysed the gut microbiomes of patients with schizophrenia at the AMHC, Anhui, China. The data compared the gut flora at the genus level among the acute, remission, and healthy groups. First, we observed an overall alteration in the diversity and abundance of bacterial communities in the gut microbiota of individuals at different stages of schizophrenia. The results of alpha estimators did not reveal alterations among the three groups, and these outcomes are consistent with those of previous studies (16, 37). The results of beta-diversity analyses demonstrated a pronounced separation of the acute group from the other two groups, these results indicated a significant divergence beta-diversity of gut microbiota among the three groups. This outcome is consistent with those reported by other studies that analysed the beta-diversity between the schizophrenia and healthy groups (38).

Many studies have reported the anomalous taxonomic in the gut microbiota between the schizophrenia and healthy groups, although the drivers of community separation varied considerably across studies (38). Many inconsistent results have also been reported for the gut microbiota at the genus level. Six genera have been found to relatively increase in schizophrenia, while five genera have been reported to decrease schizophrenia (16, 37). In our study, we found an abundance of gut bacteria at the genus level including *Succinivibrio, Haemophilus, Lactobacillus, Megasphaera, Desulfovibrio, Coprococcus, Clostridium*, and *Lachnospira* were altered during different stages of schizophrenia. Previously, these bacteria have been shown to be associated with neuropsychiatric disorders (37, 39). We integrated the effects of gut microbiota on schizophrenia and found that various genera could serve as predictors of disease progression. The ratio of abundance in specific bacterial populations was also evaluated (beta-diversity measures). The microbiota structures defined by the beta-diversity index were quantitatively discrepant at different stages of the disease. We showed that the richness and diversity of faecal microbiota, defined by beta-diversity indices, were altered in the acute, remission, and healthy groups.

A recent study reported that the intestinal microbiota is highly variable at species-level phylotypes in different populations. Some external and internal factors of the microbiota–host and microbiota–microbiota interactions may influence the composition and activity of the gut microbiota. In some instances, dysbiosis may be a normal physiological phenomenon rather than a pathophysiological condition. Therefore, the correlation between gut microbiota imbalance and disease symptoms warrants further investigation to provide more information about the mechanism of the disease (40). Furthermore, we observed that healthy individuals had a high abundance of *Coprococuus* and a low abundance of *Megasphaera, Succinivibrio, Clostridium, Veilloneaceae*, and *Megamonas*. In the remission stage of schizophrenia, the number of *Megasphaera* and *Megamonas* numbers were high. In contrast, *Haemophilus and Faecalibacterium* numbers were reduced in the acute stage of the disease. These variations suggest a transformation in the human gut microbiome at different stages of the disease. Previous studies have shown that psychotic symptom severity has a positive relationship with *Bacteroidaceae* and *Streptococcaceae* and a negative relationship with *Veilloneaceae* (16, 37). Furthermore, Nguyen et al. (37) found the abundance of Ruminococcaceae was correlated with lower severity of negative symptoms within individuals with schizophrenia. These results were not found in our study. In our study, we revealed that abundance of *Haemophilus* and *Coprococcus* was positively and negatively associated with negative symptoms, respectively. Moreover, abundance of *Haemophilus* was positively associated with symptoms of cognition, excitement, and depression based on the PANSS scale. These outcomes suggest that abundance of *Haemophilus* may be associated with a greater risk of developing psychosis in patients with schizophrenia.

This study compared the gut microbiota of participants at different stages to better understand the gut microbiota dysbiosis and the clinical features associated with schizophrenia. The current study further strengthens the argument that the gut microbiota can be a potential target for schizophrenia. The findings demonstrated dysbiosis of the gut microbiota at different stages of schizophrenia. Specifically, the abundance of *Haemophilus* and *Coprococcus* showed a positive and negative correlation with negative symptoms of schizophrenia, respectively. Prior literature have evaluate that the antipsychotic medication was not only associated with significantly reduced immunoglobulin IgA levels, IgM levels, and IgG levels, but also increased the proportion of patients using more than five antibiotic courses in a year in the schizophrenia. Obviously, IgA is involved in defence against infection at mucosal surfaces such as the upper and lower respiratory tract and gut. This might have contributed to the greater risk that the specific antibody levels below the protective range for pneumococcus and haemophilus (41). Previous studies also shown *Coprococcus* enriched among individuals with a high lifetime cardiovascular risk profile and decreased among bariatric surgery in obese patients with diabetes, suggesting that it may be a marker of coronary heart disease in patients with schizophrenia (37). To our knowledge, there have been no published reports on the significance of the ratio of *Haemophilus* and *Coprococcus* at the different stages of schizophrenia. Importantly, further analysis of the significantly altered bacterial taxa indicated that the gut microbiota is different in the diseased state, the recovery state, and healthy volunteers.

### Limitations

This study has several limitations. First, the sample size was small. Thus, the results should be considered to generate a hypothesis to clarify the relationship among different variables. Future studies should target larger-sized cohorts of psychiatric patients. Second, our study was a pilot study, hence, we could not assess causality. Third, the recruited patients were all hospitalised patients, and their diet was provided by the hospital. We did not collect detailed information about the diet. As a whole, schizophrenia patients follow a low-quality diet and are exposed to deficiencies in various nutrients that are essential for brain functioning, microbiota dysbiosis will not only affect the absorption of nutrients, but also cannot synthesise a variety of vitamins and amino acids and other nutrients. Research demonstrated if insufficient levels of vitamin D, N-3 polyunsaturated fatty acids and eicosapentaenoic acid in combination with genetic factors and a key periods during brain development, would lead to dysfunctional dopamine and serotonin activation and may be one underlying mechanism that contributes to neuropsychiatric disorders (42). The relationship between dietary intake and the gut microbiota composition should been analysed in the future studies. Finally, previous studies have shown that anti-psychotic treatment may also influence the microbiota. Although all patients were treated with second-generation anti-psychotics, a potential bias may exist in the present study.

## Conclusions

The study showed the intestinal bacteria imbalance in participants at different stages of the disease (acute vs. remission) compared to that in healthy subjects. We found that abundance of *Haemophilus* and *Coprococcus* was positively and negatively associated with the negative symptoms of schizophrenia, respectively. This could be used as an indicator of different stages of schizophrenia as well as provide new perspectives for schizophrenia research.

## Data Availability Statement

The data that support the findings of this study have been deposited in the NCBI (https://submit.ncbi.nlm.nih.gov/) with bioproject number (PRJAN750612).

## Ethics Statement

The study was approved by the Medical Ethics Committee of the Anhui Mental Health Centre (AMHC). All participants provided written consent prior to study participation in accordance with the principles of the Declaration of Helsinki. The trial registration number was chiCTR1800019343. The patients/participants provided their written informed consent to participate in this study.

## Author Contributions

XZ and JC were responsible for study design and manuscript editing. CZ, MZ, and UA were responsible for literature searches, statistical analyses, and manuscript writing. QX, C, JY, and KW were responsible for clinical-scale assessment data collection. ZW, LY, and YC were responsible for clinical blood data collection. All authors have contributed to and have approved the final manuscript.

## Conflict of Interest

The authors declare that the research was conducted in the absence of any commercial or financial relationships that could be construed as a potential conflict of interest.

## Publisher's Note

All claims expressed in this article are solely those of the authors and do not necessarily represent those of their affiliated organizations, or those of the publisher, the editors and the reviewers. Any product that may be evaluated in this article, or claim that may be made by its manufacturer, is not guaranteed or endorsed by the publisher.

## Supplementary Material

Supplementary Figure 1 Results of the alpha-diversity of the faecal microbial communities in the acute stage group, Remission stage group and Healthy control groups. Six alpha metrics of Chao, Ace, Shannon's diversity, Simpson's diversity, Observed species and Good's coverage indices were calculated, The *P*-values of those six metrics were all >0.05. Alpha-diversity was no difference among the three groups.
