# Supplementary material for: Association Between Abundance of Haemophilus in the Gut Microbiota and Negative Symptoms of Schizophrenia
Source: Front Psychiatry. 2021 Jul 30;12:685910. doi: 10.3389/fpsyt.2021.685910 (PMC8362742; doi:10.3389/fpsyt.2021.685910)

Supplementary 1, results of the alpha-diversity of the fecal microbial communities in the acute stage group, Remission stage group and Healthy control groups. Six alpha metrics of Chao, Ace, Shannon's diversity, Simpson's diversity, Observed species and Good's coverage indices were calculated, The P-values of those six metrics were all＞0.05, Alpha-diversity was no difference among the three groups.

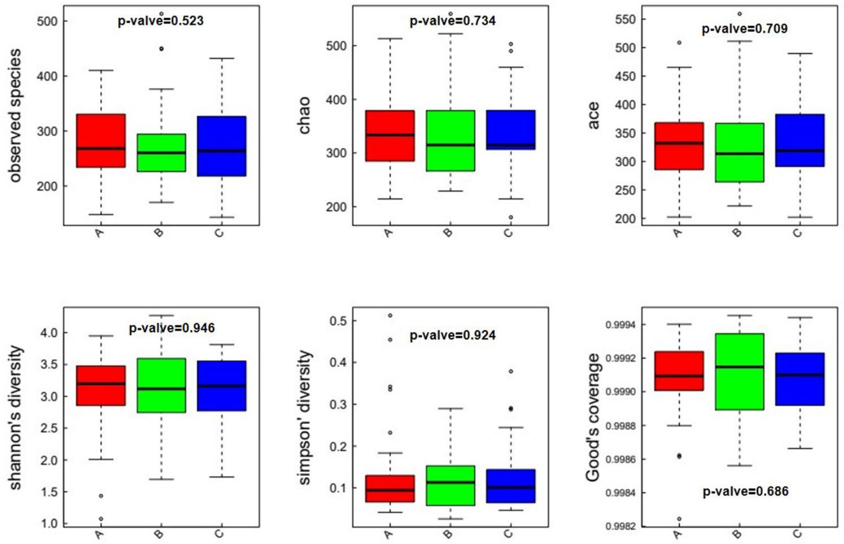

Supplement: Supplementary Figure 1 — Results of the alpha-diversity of the faecal microbial communities in the acute stage group, Remission stage group and Healthy control groups. Six alpha metrics of Chao, Ace, Shannon's diversity, Simpson's diversity, Observed species and Good's coverage indices were calculated, The P-values of those six metrics were all >0.05. Alpha-diversity was no difference among the three groups. [file Image_1.pdf]
